# Causes of Death Among Prostate Cancer Patients Aged 40 Years and Older in the United States

**DOI:** 10.3389/fonc.2022.914875

**Published:** 2022-07-01

**Authors:** Yuzhong Ye, Yongqiang Zheng, Qi Miao, Hailong Ruan, Xiaoping Zhang

**Affiliations:** ^1^ Department of Urology, Union Hospital, Tongji Medical College, Huazhong University of Science and Technology, Wuhan, China; ^2^ State Key Laboratory of Oncology in South China, Sun Yat-sen University Cancer Center, Sun Yat-sen University, Guangzhou, China

**Keywords:** prostate cancer, cause of death, non-cancer deaths, cardiovascular diseases, chronic obstructive pulmonary disease and allied cond, Surveillance Epidemiology and End Results database

## Abstract

**Purpose:**

Little is known about the detailed spectrum of the cause of death associated with prostate cancer (PCa). This study systematically characterized the cause of death among patients with PCa.

**Methods:**

Patients aged 40 years and older with primary PCa were identified from the Surveillance, Epidemiology, and End Results program. Mortality rates were estimated. Standardized mortality ratios (SMRs) of non-cancer deaths were calculated to evaluate the risk of death and to compare with the cancer-free population.

**Results:**

This study included 1,170,489 patients with PCa. There were 501,262 deaths, of which 27.4% were due to PCa and 57.0% were due to non-cancer causes. Non-cancer deaths increased over time from 1975 to 2016, and index cancer death decreased continually. The risk of non-cancer deaths was 1.45 times (SMR, 1.45; 95% confidence interval [CI], 1.45–1.46) that of the general population. Cardiovascular disease was the most common non-cancer cause of death, accounting for 30.2% of all deaths among PCa patients. Alzheimer’s disease (SMR, 3.92; 95% CI, 3.85–4.00) had the highest risk of death. The mortality rate and SMR of non-cancer deaths increased with increased follow-up after diagnosis.

**Conclusion:**

Instead of the index cancer, non-cancer comorbidities were the leading cause of death among patients with PCa, and the risk of non-cancer deaths was much higher than among the general population. Clinicians and researchers should be aware of this trend to conduct timely and targeted interventions.

## Introduction

Prostate cancer (PCa) represents the most common cancer in American men, with an estimated 248,530 new cases in 2021 (1). Since 1990, the United States has been in the era of prostate-specific antigen (PSA) screening (2). The “lead time effect” of PSA screening has led to downward stage migration, with an increased proportion of localized PCa (mostly asymptomatic) and a decreased proportion of metastatic PCa being diagnosed (3). The latest data show that the 5-year survival rate for PCa is close to 100%, which means that most patients have a long survival period (4). It is well known that PCa is an age-related disease, with a median age at diagnosis of 66 years (5). As the population ages, the distribution of causes of death in this patient population will become the focus of epidemiologists and doctors (6, 7).

The dangers of non-cancer comorbidities are being increasingly noticed by clinicians since they are important causes of death in cancer patients (8). Some non-cancer comorbidities, including cardiovascular diseases (9), infectious diseases (10), and accidents (11), were reported to be major threats to the health of cancer patients. Several previous PCa studies using data from the Danish Cancer Registry, nationwide Swedish Cancer Registry, and the US Surveillance, Epidemiology, and End Results Program (SEER) suggest that cardiovascular diseases, cerebrovascular diseases, and other age-related diseases were common the causes of non-cancer deaths. However, the scope of these studies was often limited, and they only covered a few common diseases (8, 12–14). Therefore, it is necessary to draw a detailed spectrum of cause of death in PCa patients, which could help researchers and clinicians recognize the significant impacts of various non-cancer causes of death in PCa patients.

This study aimed to comprehensively investigate the distribution of cause of death in PCa survivors to provide constructive evidence for the long-term health management of patients and the optimization of survival and quality of life.

## Materials and Methods

### Study Population and Data Source

This retrospective cohort study was conducted using data from the SEER program (http://seer.cancer.gov). The SEER program covers approximately 34.6% of the US population and collects data on the demographics, tumor morphology, and stage at diagnosis, anatomic site, therapy, follow-up data, and socioeconomic status of cancer patients.

**Figure 1:**
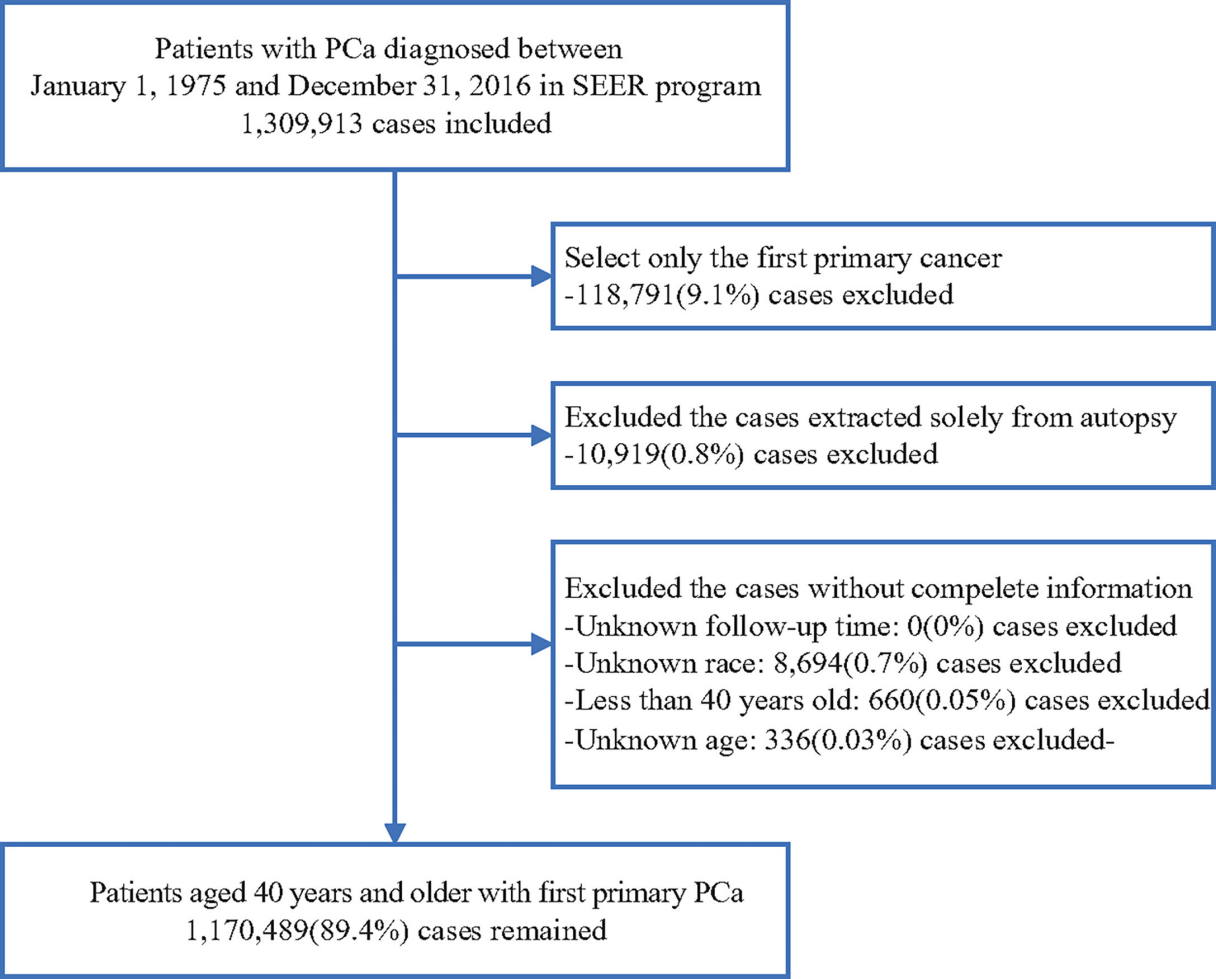
Flow chart of case inclusion and exclusion criteria.

All PCa patients (site code C61.9) diagnosed between 1975 and 2016 were retrieved from the SEER 18 database (2019 submission) (N = 1,170,489) (15). Only patients aged 40 years and older with a primary diagnosis of PCa were included. Patients whose diagnosis was only extracted from the death certificate and whose follow-up information was incomplete were excluded (see **Figure 1** for details). For comparison, we extracted the age-specific mortality data of the general US population between 1975 and 2016 from the SEER program (16).

The Institutional Review Boards of the Union Hospital of the Tongji Medical College waived the institutional review board approval for the data that were obtained from the SEER database, as the study did not directly involve human subjects.

### Definition of Variables

The follow-up period was defined as the date from the diagnosis of PCa to the date of death from any cause, the end of the study (31 December 2016), or early withdrawal. We extracted patients’ demographic and clinical information, including age, sex, race, marital status, age at diagnosis, PSA level, Gleason score, anatomic site, survival time, stage at diagnosis, and surgery from the SEER database. The causes of death were classified into three groups: 1. death from the index cancer, 2. death from a non-index cancer (i.e., second or subsequent primary cancers), and 3. death from non-cancer causes. According to the SEER cause-specific death classification variable, non-cancer deaths were categorized into 26 groups (17). Non-cancer deaths were further classified into seven major categories: cardiovascular diseases, infectious diseases, respiratory diseases, kidney diseases, gastrointestinal and liver diseases, external injuries, and other causes. Deaths from tumors with histology of “*in situ*, benign, or unknown behavior neoplasm” were treated as non-index cancer deaths rather than non-cancer deaths in our study. For patients who died within 1 month after diagnosis, the SEER project recorded their survival months as 0 months. Given that the survival time of this group of patients ranged from 0 to 29 days, we converted their survival time to the mean of this duration, namely, 0.5 months.

### Statistical Analysis

We calculated the mortality of the three groups among all PCa patients. For non-cancer causes, mortality rates were calculated as the ratio of the death toll of PCa patients to that of the general population. The calculation method of standardized mortality ratios (SMRs) and 95% confidence intervals (CIs) of non-cancer causes was as previously described (18–20). SMRs were calculated as the ratio of the observed number of deaths in cancer patients to the expected number of deaths in the general population, which had a similar demographical structure of sex, age, race, and calendar year. For age and calendar year, the value at cancer diagnosis was used, and 5-year categories were created in the course of standardization. The 95% CIs are calculated using Poisson distribution (18, 21). The SMRs were not available for cancer-related deaths, because they were based on an assumption that regards the general population as a cancer-free cohort.

Data on deaths were extracted from SEER 9 registries databases which continually code death trends from diverse causes by calendar year of cancer diagnosis from 1975 to 2006. When analyzing trends of cause of death by year of diagnosis, we limited follow-up to up to 10 years to avoid bias caused by the variations in lengths of follow-up for patients diagnosed in different periods. Patients diagnosed in 2007–2016 were excluded from this analysis due to insufficient follow-up time.

R version 3.52 statistical software and SEER*Stat software version 8.3.6 were used for data analysis (22, 23). All hypothesis tests were two-sided, and P-value < 0.05 was set as the significance level.

## Results

Our study included 1,170,489 PCa patients diagnosed between 1975 and 2016, with a median follow-up time of 6.9 years (range: 0–41.7 years). The demographic and tumor-related features of the study participants are reported in **Table 1**. The mean age at diagnosis was 67.7 years, and the majority of the patients were elderly (>60 years old, 79.6%) and white (79.0%) (**Table 1**).

**Table 1:** Baseline characteristics of patients diagnosed with prostate cancer between 1975 and 2016 in SEER 18 registries.

Characteristics	No. of patients with cancer (%)	Total person years of follow-up	No. of deaths from all causes (%)	Death from non-cancer causes		
				No. of observed deaths ^a^ (%)	No. of expected deaths ^a^	SMR ^b^ (95% CI)
All patients	1,170,489 (100.0%)	9,149,673	501,262 (100.0%)	285,738 (100.0%)	196,441.0	1.45 (1.45-1.46)
Age						
40-59	239,098 (20.4%)	2,148,041	40,227 (8.0%)	16,143 (5.6%)	13,263.6	1.22 (1.20-1.24)
60-79	798,356 (68.2%)	6,414,766	351,946 (70.2%)	201,771 (70.6%)	131,604.3	1.53 (1.53-1.54)
80+	133,035 (11.4%)	586,866	109,089 (21.8%)	67,824 (23.7%)	51,573.1	1.32 (1.31-1.33)
Race						
White	924,461 (79.0%)	7,420,008	408,843 (81.6%)	235,106 (82.3%)	160,285.8	1.47 (1.46-1.47)
Black	163,786 (14.0%)	1,166,480	68,452 (13.7%)	36,784 (12.9%)	27,710.9	1.33 (1.31-1.34)
Other	82,242 (7.0%)	563,186	23,967 (4.8%)	13,848 (4.8%)	8,444.3	1.64 (1.61-1.67)
Year						
1975-1989	108,993 (9.3%)	878,388	107,078 (21.4%)	57,709 (20.2%)	30,918.3	1.87 (1.85-1.88)
1990-1999	212,482 (18.2%)	2,451,915	164,300 (32.8%)	99,786 (34.9%)	58,878.3	1.69 (1.68-1.71)
2000-2009	516,940 (44.2%)	4,755,209	195,906 (39.1%)	113,298 (39.7%)	89,503.8	1.27 (1.26-1.27)
2010-2016	332,074 (28.4%)	1,064,162	33,978 (6.8%)	14,945 (5.2%)	17,140.6	0.87 (0.86-0.89)
Marital status						
Married	782,158 (66.8%)	6,597,421	328,325 (65.5%)	185,454 (64.9%)	134,770.6	1.38 (1.37-1.38)
Unmarried	253,886 (21.7%)	1,717,954	126,851 (25.3%)	71,623 (25.1%)	41,570.8	1.72 (1.71-1.74)
Unknown	134,445 (11.5%)	834,298	46,086 (9.2%)	28,661 (10.0%)	20,099.6	1.43 (1.41-1.44)
PSA						
< 10	391,707 (33.5%)	2,395,091	48,826 (9.7%)	30,032 (10.5%)	34,543.3	0.87 (0.86-0.88)
10-20	85,112 (7.3%)	463,777	19,494 (3.9%)	11,359 (4.0%)	9,671.5	1.17 (1.15-1.20)
> 20	70,925 (6.1%)	281,066	31,630 (6.3%)	10,740 (3.8%)	7,266.5	1.48 (1.45-1.51)
Unknown	622,745 (53.2%)	6,009,739	401,312 (80.1%)	233,607 (81.8%)	144,959.7	1.61 (1.61-1.62)
Gleason score						
2-5	5,600 (0.5%)	49,325	1,873 (0.4%)	1,333 (0.5%)	989.3	1.35 (1.28-1.42)
6	126,179 (10.8%)	1,121,485	26,792 (5.3%)	18,262 (6.4%)	17,489.0	1.04 (1.03-1.06)
3+4	76,582 (6.5%)	658,929	17,722 (3.5%)	11,256 (3.9%)	10,554.5	1.07 (1.05-1.09)
4+3	29,491 (2.5%)	239,212	9,610 (1.9%)	5,522 (1.9%)	4,670.9	1.18 (1.15-1.21)
8-10	42,217 (3.6%)	283,901	22,317 (4.5%)	9,210 (3.2%)	6,804.3	1.35 (1.33-1.38)
Unknown	890,420 (76.1%)	6,796,822	422,948 (84.4%)	240,155 (84.0%)	155,933.1	1.54 (1.53-1.55)
Stage						
In situ	162 (0.0%)	1,210	88 (0.0%)	63 (0.0%)	37.3	1.69 (1.32-2.16)
Localized/regional	826,079 (70.6%)	6,741,826	246,099 (49.1%)	155,199 (54.3%)	123,907.0	1.25 (1.25-1.26)
Distant	43,866 (3.7%)	123,637	35,146 (7.0%)	6,730 (2.4%)	3,820.7	1.76 (1.72-1.80)
Unstaged	300,382 (25.7%)	2,283,000	219,929 (43.9%)	123,746 (43.3%)	68,676.0	1.80 (1.79-1.81)
Surgery						
Yes	498,498 (42.6%)	4,484,719	192,301 (38.4%)	113,093 (39.6%)	79,056.0	1.43 (1.42-1.44)
No	651,498 (55.7%)	4,543,600	298,605 (59.6%)	167,762 (58.7%)	114,302.4	1.47 (1.46-1.47)
Unknown	20,493 (1.8%)	121,354	10,356 (2.1%)	4,883 (1.7%)	3,082.6	1.58 (1.54-1.63)

SMR, standardized mortality ratios; CI, confidence interval; PSA, prostate-specific antigen.

a Observed deaths represents the number of deaths from certain causes in cancer patients; Expected number of deaths represents the number of people who died from the same causes in the general population with a similar distribution of age at diagnosis, sex, race, and calendar year.

b The SMRs were estimated as the ratios of observed to expected number of deaths.

### Cause of Death Among PCa Patients

Among the 501,262 patients who died during follow-up, 27.4% (n = 137,188) died of PCa, 15.6% (n = 78,336) died of non-index cancers, and 57% (n = 285,764) died of non-cancer causes. Cardiovascular diseases caused the majority of non-cancer deaths, accounting for 30.2% of all deaths in patients with PCa, followed by respiratory diseases (4.2%) and infectious diseases (3.9%) (**Table 2**).

**Table 2:** Cause of death for patients diagnosed with prostate cancers between 1975 and 2016 in SEER 18 registries.

Causes of death	Patients with prostate cancer		General population		SMR ^a^ (95% CI)
	No. of observed deaths (%)	Mortality rates (per 100,000 person-years)	No. of expected deaths (%)	Mortality rates (per 100,000 person-years)	
All causes of death	501,288 (100.0%)	5,476.8			
Prostate cancer	137,188 (27.4%)	1,499.4			
Other cancers	78,336 (15.6%)	856.2			
Non-cancer causes	285,764 (57.0%)	3,121.2	196,452.0	2,145.7	1.45 (1.45-1.46)
Infectious	19,362 (3.9%)	211.6	12,703.7	138.8	1.52 (1.50-1.55)
Pneumonia and Influenza	12,410 (2.5%)	135.6	7,304.6	79.8	1.70 (1.67-1.73)
Tuberculosis	126 (0.0%)	1.4	184.0	2.0	0.68 (0.57-0.82)
Septicemia	4,535 (0.9%)	49.6	3,047.6	33.3	1.49 (1.45-1.53)
Other infectious and parasitic diseases including HIV	2,291 (0.5%)	25.0	2,163.4	23.6	1.06 (1.02-1.10)
Cardiovascular diseases	151,555 (30.2%)	1,656.4	111,505.4	1,218.7	1.36 (1.35-1.37)
Diseases of heart	117,857 (23.5%)	1,288.1	89,072.7	973.5	1.32 (1.32-1.33)
Hypertension without heart disease	3,916 (0.8%)	42.8	1,759.1	19.2	2.23 (2.16-2.30)
Cerebrovascular diseases	23,516 (4.7%)	257.0	15,577.6	170.3	1.51 (1.49-1.53)
Atherosclerosis	2,407 (0.5%)	26.3	1,438.1	15.7	1.67 (1.61-1.74)
Aortic aneurysm and dissection	2,425 (0.5%)	26.5	2,547.2	27.8	0.95 (0.91-0.99)
Other diseases of arteries, arterioles, capillaries	1,434 (0.3%)	15.7	1,110.4	12.1	1.29 (1.23-1.36)
Respiratory diseases	21,194 (4.2%)	231.6	15,927.2	174.1	1.33 (1.31-1.35)
Chronic obstructive pulmonary disease and allied cond	21,194 (4.2%)	231.6	15,927.2	174.1	1.33 (1.31-1.35)
Gastrointestinal diseases	3,047 (0.6%)	33.3	4,105.9	44.9	0.74 (0.72-0.77)
Stomach and duodenal ulcers	821 (0.2%)	9.0	598.7	6.5	1.37 (1.28-1.47)
Chronic liver disease and cirrhosis	2,226 (0.4%)	24.3	3,507.1	38.3	0.63 (0.61-0.66)
Renal diseases	6,622 (1.3%)	72.4	4,104.2	44.9	1.61 (1.58-1.65)
Nephritis, nephrotic syndrome and nephrosis	6,622 (1.3%)	72.4	4,104.2	44.9	1.61 (1.58-1.65)
External injuries	12,302 (2.5%)	134.5	9,724.5	106.3	1.27 (1.24-1.29)
Accidents and adverse effects	8,990 (1.8%)	98.3	6,761.7	73.9	1.33 (1.30-1.36)
Suicide and self-Inflicted injury	3,042 (0.6%)	33.2	2,450.8	26.8	1.24 (1.20-1.29)
Homicide and legal intervention	270 (0.1%)	3.0	510.3	5.6	0.53 (0.47-0.60)
Other non-cancer causes	71,656 (14.3%)	783.2	38,370.0	419.4	1.87 (1.85-1.88)
Diabetes mellitus	9,741 (1.9%)	106.5	7,830.2	85.6	1.24 (1.22-1.27)
Alzheimers	10,634 (2.1%)	116.2	2,710.8	29.6	3.92 (3.85-4.00)
Congenital anomalies	289 (0.1%)	3.2	265.3	2.9	1.09 (0.97-1.22)
Symptoms, signs and ill-defined conditions	3,138 (0.6%)	34.3	2,156.9	23.6	1.45 (1.40-1.51)
Other cause of death	47,854 (9.5%)	523.0	25,400.8	277.6	1.88 (1.87-1.90)

a SMR is estimated as the ratio of observed deaths to expected deaths. In terms of age, sex, and year of diagnosis, the SMR reference population was the specific category in the US subpopulation from 1975 through 2016. For race/ethnicity, the specific race/ethnicity codes (white, black, American Indian or Alaska Native, and Asianor Pacific Islander) were used. The calculating of SMR is based on the hypothesis that the general population is cancer-free, thus the expected deaths and SMR for prostate cancer and other cancers cannot be calculated.

Among patients who died of non-cancer causes, the mortality rate in patients with PCa was 1.45 times that of the general population (SMR, 1.45; 95% CI, 1.45–1.46). Patients diagnosed before the 1990s, those 60–79 years old, those of non-black ethnicity, unmarried patients, patients with a PSA between 10 and 20 ng/ml, and patients with distant type were more likely to die from noncancer causes than the general population. Except for patients diagnosed between 2010 and 2016 and those with a PSA <10 ng/ml, the SMRs of non-cancer deaths in all subgroups were >1 and statistically significant (**Table 1**).

**Figure 2:**
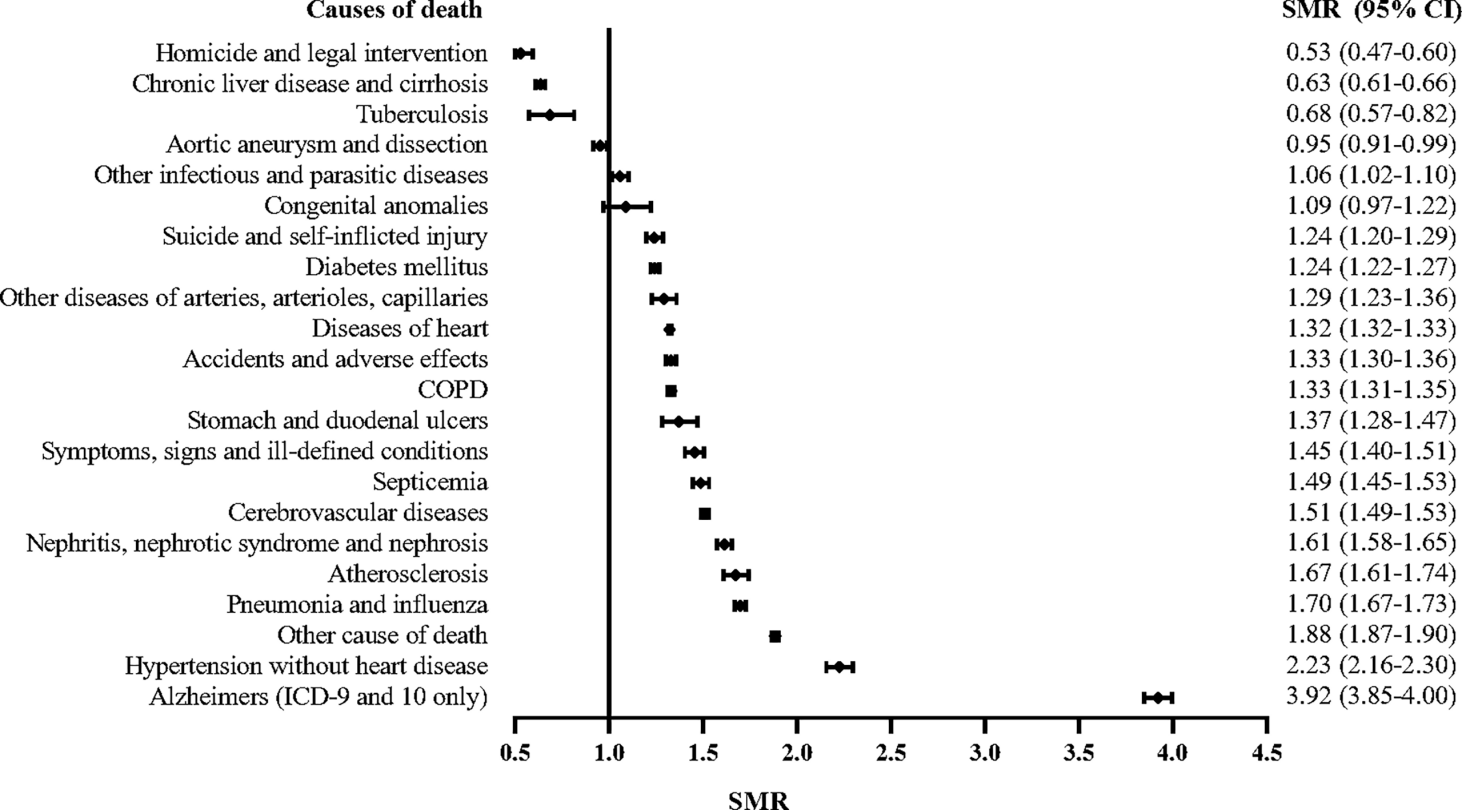
Forest plot showing the SMRs of different non-cancer of death in ranked order.

The mortality of cardiovascular diseases was 36% higher in PCa patients than in the general population (SMR, 1.36; 95% CI, 1.35–1.37). The SMRs of infectious diseases and respiratory diseases in PCa patients were 1.52 (95% CI, 1.50–1.55), and 1.33 (95% CI, 1.31–1.35), respectively. We also examined more detailed causes of death and found that the risk were highest for Alzheimer’s disease and hypertension, which had SMRs of 3.92 (95% CI, 3.85–4.00) and 2.23 (95% CI, 2.16–2.30), respectively (**Table 2**, **Figure 2**).

### Trends of Cause of Death Among PCa Patients by Year of Cancer Diagnosis

**Figure 3:**
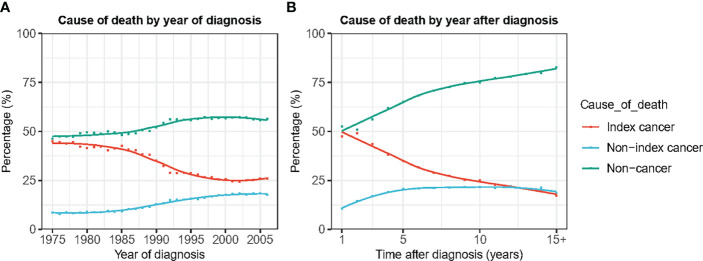
Cause of death by year of death and time after diagnosis. **(A)** Cause of death by year of death. **(B)** Cause of death by time after diagnosis.

In patients diagnosed in the 1970s, the gap between the proportion of deaths from PCa and non-cancer causes was not very large. Among patients diagnosed in 1975, 45.0% and 46.4% died of PCa and non-cancer causes, respectively (**Figure 3**). However, the proportion of deaths caused by PCa showed a rapid downward trend with the year of diagnosis, whereas the proportion of non-cancer deaths showed a clear upward trend. Among patients diagnosed in 2006, primary cancer accounted for only 17.6% of all deaths, compared with 56.4% of deaths from non-cancer causes (**Figure 3A**).

### Trends of Cause of Death Among PCa Patients by Time After Cancer Diagnosis

**Figure 4:**
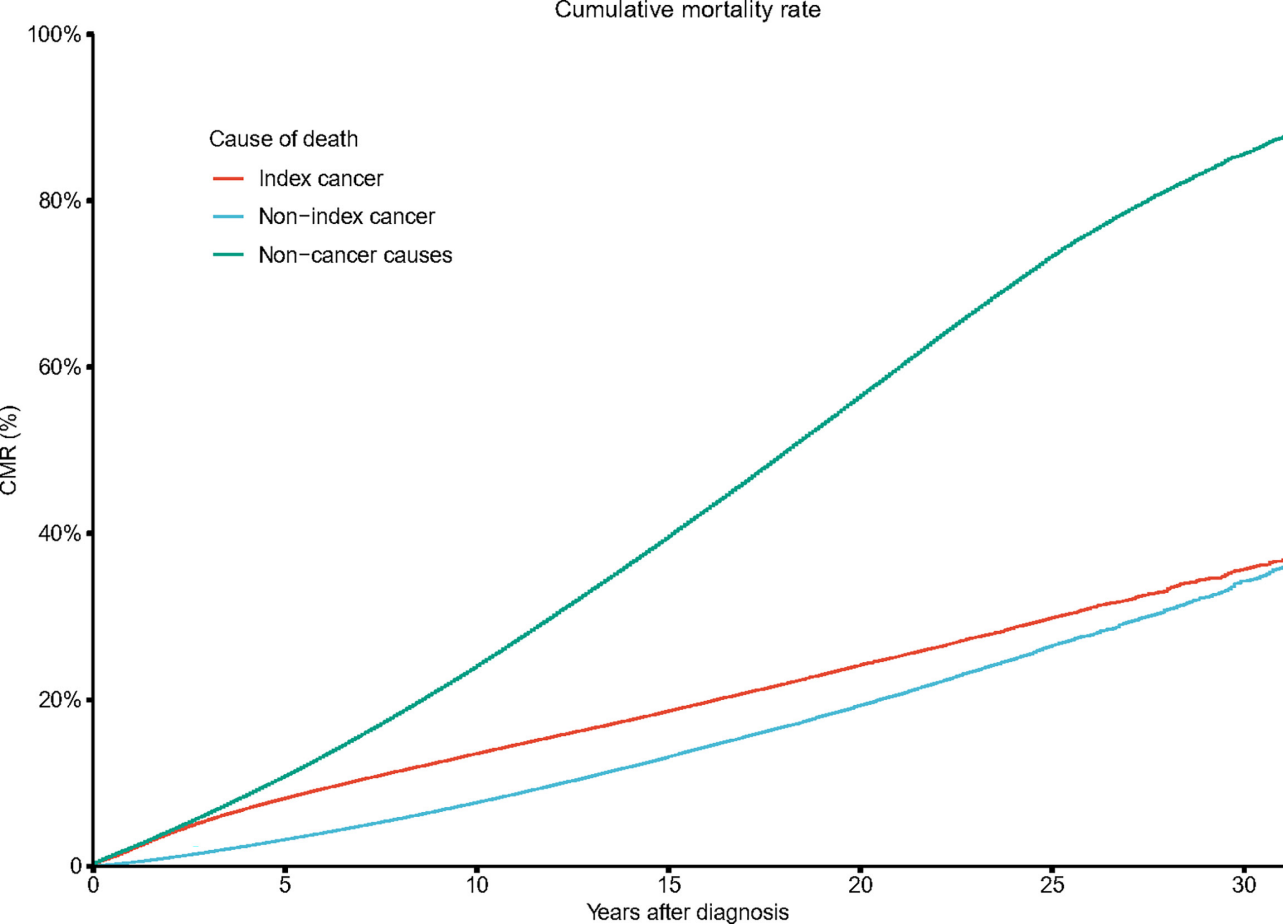
Cumulative mortality rate (SMR) by time after diagnosis.

Non-cancer deaths accounted for the highest proportion of deaths throughout the follow-up period. As the follow-up time increased, the proportion of deaths from PCa gradually decreased, and the proportion of non-cancer deaths gradually increased. Beginning in the second year after diagnosis, the gap in the proportion of PCa-specific deaths and non-cancer deaths continued to widen (**Figure 3B**). After the 10th year, non-cancer deaths accounted for 67.1% of all deaths, which was four times that of PCa-specific deaths (**Figure 3B**). The cumulative mortality rate (CMR) of the index cancer and non-index cancer increased slowly by time after diagnosis. The 5-year and 10-year CMRs of index cancer were 8.1% and 13.5%, respectively. The 5-year and 10-year CMRs of non-index cancer were 3.2% and 7.7%, separately. In contrast, the CMR of non-cancer causes increased rapidly, and the 5-year and 10-year CMR were 10.7% and 23.9%, respectively (**Figure 4**).

The SMRs of non-cancer deaths increased with prolonged survival time, and it was highest more than 10 years after diagnosis, with an SMR of 2.98 (95% CI, 2.96–3.00). This increasing trend was observed for most causes of death and was highest for Alzheimer’s disease. However, the SMRs of suicide and gastroduodenal ulcer death were significantly higher in the first year after diagnosis than 1–5 years after diagnosis (**Table 3**).

**Table 3:** Cause of death among patients diagnosed with prostate cancer between 1975 and 2016 in SEER 18 registries by time from cancer diagnosis.

Cause of death	Time from cancer diagnosis							
	1 years		1-5 years		5-10 years		10+ years	
	No. of observed deaths (%)	SMR (95% CI)	No. of observed deaths (%)	SMR (95% CI)	No. of observed deaths (%)	SMR (95% CI)	No. of observed deaths (%)	SMR (95% CI)
All causes of death	51,372 (100.0%)	NA	164,555 (100.0%)	NA	144,956 (100.0%)	NA	140,379 (100.0%)	NA
Prostate cancer	22,062 (42.9%)	NA	58,887 (35.8%)	NA	33,391 (23.0%)	NA	22,848 (16.3%)	NA
Other cancers	4,905 (9.5%)	NA	24,552 (14.9%)	NA	25,475 (17.6%)	NA	23,404 (16.7%)	NA
Non-cancer causes	24,405 (47.5%)	0.95 (0.94-0.96)	81,116 (49.3%)	0.98 (0.97-0.98)	86,090 (59.4%)	1.53 (1.52-1.54)	94,127 (67.1%)	2.98 (2.96-3.00)
Infectious	1,696 (3.3%)	0.98 (0.93-1.02)	5,743 (3.5%)	1.04 (1.02-1.07)	5,811 (4.0%)	1.62 (1.58-1.66)	6,112 (4.4%)	3.26 (3.18-3.35)
Pneumonia and Influenza	1,047 (2.0%)	1.00 (0.94-1.07)	3,677 (2.2%)	1.14 (1.11-1.18)	3,740 (2.6%)	1.86 (1.80-1.92)	3,946 (2.8%)	3.80 (3.68-3.92)
Tuberculosis	25 (0.0%)	1.14 (0.77-1.68)	41 (0.0%)	0.57 (0.42-0.77)	32 (0.0%)	0.61 (0.43-0.86)	28 (0.0%)	0.75 (0.52-1.09)
Septicemia	405 (0.8%)	0.98 (0.89-1.08)	1,328 (0.8%)	0.99 (0.94-1.05)	1,377 (0.9%)	1.56 (1.48-1.64)	1,425 (1.0%)	3.43 (3.26-3.61)
Other infectious and parasitic diseases including HIV	219 (0.4%)	0.85 (0.75-0.97)	697 (0.4%)	0.79 (0.73-0.85)	662 (0.5%)	1.03 (0.95-1.11)	713 (0.5%)	1.87 (1.74-2.01)
Cardiovascular diseases	14,715 (28.6%)	1.03 (1.01-1.04)	45,385 (27.6%)	0.98 (0.97-0.99)	45,148 (31.1%)	1.42 (1.40-1.43)	46,307 (33.0%)	2.46 (2.44-2.48)
Diseases of heart	11,676 (22.7%)	1.03 (1.01-1.05)	35,602 (21.6%)	0.97 (0.96-0.98)	34,896 (24.1%)	1.37 (1.35-1.38)	35,683 (25.4%)	2.32 (2.29-2.34)
Hypertension without heart disease	273 (0.5%)	1.08 (0.96-1.22)	988 (0.6%)	1.25 (1.17-1.33)	1,212 (0.8%)	2.45 (2.32-2.59)	1,443 (1.0%)	6.48 (6.16-6.83)
Cerebrovascular diseases	2,082 (4.1%)	0.99 (0.94-1.03)	6,821 (4.1%)	1.02 (1.00-1.04)	7,240 (5.0%)	1.64 (1.60-1.68)	7,373 (5.3%)	3.12 (3.05-3.20)
Atherosclerosis	273 (0.5%)	1.28 (1.13-1.44)	756 (0.5%)	1.19 (1.11-1.28)	692 (0.5%)	1.80 (1.67-1.93)	686 (0.5%)	3.39 (3.15-3.65)
Aortic aneurysm and dissection	280 (0.5%)	0.94 (0.83-1.05)	813 (0.5%)	0.80 (0.75-0.86)	704 (0.5%)	0.93 (0.87-1.01)	628 (0.4%)	1.30 (1.20-1.41)
Other diseases of arteries, arterioles, capillaries	131 (0.3%)	0.91 (0.77-1.08)	405 (0.2%)	0.87 (0.79-0.96)	404 (0.3%)	1.26 (1.15-1.39)	494 (0.4%)	2.72 (2.49-2.97)
Respiratory diseases	1,682 (3.3%)	0.83 (0.79-0.87)	6,333 (3.8%)	0.94 (0.92-0.97)	6,679 (4.6%)	1.44 (1.40-1.47)	6,500 (4.6%)	2.58 (2.52-2.64)
Chronic obstructive pulmonary disease and allied cond	1,682 (3.3%)	0.83 (0.79-0.87)	6,333 (3.8%)	0.94 (0.92-0.97)	6,679 (4.6%)	1.44 (1.40-1.47)	6,500 (4.6%)	2.58 (2.52-2.64)
Gastrointestinal diseases	329 (0.6%)	0.71 (0.64-0.79)	1,155 (0.7%)	0.72 (0.68-0.76)	856 (0.6%)	0.71 (0.66-0.76)	707 (0.5%)	0.86 (0.80-0.92)
Stomach and duodenal ulcers	107 (0.2%)	1.42 (1.18-1.72)	279 (0.2%)	1.14 (1.01-1.28)	231 (0.2%)	1.36 (1.19-1.54)	204 (0.1%)	1.88 (1.64-2.16)
Chronic liver disease and cirrhosis	222 (0.4%)	0.57 (0.50-0.65)	876 (0.5%)	0.64 (0.60-0.69)	625 (0.4%)	0.60 (0.56-0.65)	503 (0.4%)	0.70 (0.64-0.77)
Renal diseases	461 (0.9%)	0.79 (0.73-0.87)	1,673 (1.0%)	0.91 (0.87-0.96)	2,052 (1.4%)	1.75 (1.68-1.83)	2,436 (1.7%)	4.73 (4.54-4.92)
Nephritis, nephrotic syndrome and nephrosis	461 (0.9%)	0.79 (0.73-0.87)	1,673 (1.0%)	0.91 (0.87-0.96)	2,052 (1.4%)	1.75 (1.68-1.83)	2,436 (1.7%)	4.73 (4.54-4.92)
External injuries	1,139 (2.2%)	0.94 (0.89-1.00)	3,884 (2.4%)	0.97 (0.94-1.00)	3,695 (2.5%)	1.31 (1.27-1.35)	3,584 (2.6%)	2.14 (2.07-2.21)
Accidents and adverse effects	708 (1.4%)	0.82 (0.76-0.88)	2,724 (1.7%)	0.96 (0.92-0.99)	2,688 (1.9%)	1.38 (1.33-1.43)	2,870 (2.0%)	2.61 (2.52-2.71)
Suicide and self-Inflicted injury	382 (0.7%)	1.35 (1.22-1.49)	1,054 (0.6%)	1.08 (1.01-1.14)	931 (0.6%)	1.29 (1.21-1.37)	675 (0.5%)	1.46 (1.35-1.57)
Homicide and legal intervention	49 (0.1%)	0.91 (0.69-1.20)	106 (0.1%)	0.56 (0.46-0.67)	76 (0.1%)	0.50 (0.40-0.63)	39 (0.0%)	0.34 (0.25-0.47)
Other non-cancer causes	4,383 (8.5%)	0.82 (0.80-0.85)	16,943 (10.3%)	1.01 (0.99-1.02)	21,849 (15.1%)	2.01 (1.99-2.04)	28,481 (20.3%)	5.32 (5.26-5.38)
Diabetes mellitus	757 (1.5%)	0.77 (0.72-0.83)	2,810 (1.7%)	0.86 (0.82-0.89)	3,086 (2.1%)	1.33 (1.28-1.38)	3,088 (2.2%)	2.48 (2.40-2.57)
Alzheimers	311 (0.6%)	0.69 (0.62-0.77)	1,754 (1.1%)	1.33 (1.27-1.39)	3,233 (2.2%)	4.53 (4.38-4.69)	5,336 (3.8%)	23.49 (22.87-24.13)
Congenital anomalies	32 (0.1%)	1.04 (0.74-1.47)	82 (0.0%)	0.78 (0.63-0.97)	87 (0.1%)	1.11 (0.90-1.37)	88 (0.1%)	1.74 (1.41-2.14)
Symptoms, signs and ill-defined conditions	210 (0.4%)	0.72 (0.63-0.82)	858 (0.5%)	0.93 (0.87-1.00)	908 (0.6%)	1.51 (1.41-1.61)	1,162 (0.8%)	3.40 (3.21-3.60)
Other cause of death	3,073 (6.0%)	0.86 (0.83-0.89)	11,439 (7.0%)	1.02 (1.00-1.04)	14,535 (10.0%)	2.03 (2.00-2.07)	18,807 (13.4%)	5.39 (5.31-5.46)

NA, Not Applicable.

## Discussion

In this study, we conducted a comprehensive analysis of the cause of death spectrum of 1.17 million American PCa patients. The results showed that non-cancer deaths in PCa patients exceeded cancer-related deaths. The risk of non-cancer deaths was nearly 1.5 times that of the general population.

As described above, except for patients diagnosed between 2010 and 2016 and those with a PSA <10 ng/ml, the SMRs of non-cancer deaths in all subgroups were >1 and statistically significant. Patients diagnosed between 2010 and 2016 are still in the first 5 years of the follow-up period, which is the dominant period of PCa-specific death; therefore, the follow-up may be insufficient to assess non-PCa death. Clinical characteristics such as age at diagnosis, tumor stage, and Gleason score had a great influence on the risk of non-cancer deaths. Notably, a PSA >20 ng/ml that was associated with poorer outcomes in terms of non-cancer deaths suggests that patients with more advanced PCa were more likely to die from non-cancer causes. As a prostate-specific serum marker, PSA levels can distinguish different cancer stages. The PSA of patients with metastatic PCa is higher than that of with localized PCa, which in turn is higher than that of men without PCa. In addition, patients with higher pretreatment PSA had a higher risk of recurrence (24). According to the guidelines, more conservative treatment measures may be adopted for localized PCa, represented by the active surveillance strategy. For advanced PCa, more aggressive treatment measures are taken, represented by androgen deprivation therapy (ADT) for metastatic PCa and docetaxel and prednisolone for castration-resistant PCa (25, 26). Potential side effects from treatment may be one of the reasons for this phenomenon (27, 28).

The spectrum of cause of death also changed more significantly with increased follow-up time. The risk of non-cancer deaths continued to rise with the extension of survival time. After the 10th year, non-cancer deaths accounted for 67% of all deaths, which was four times that of PCa-specific deaths. Specifically, cardiovascular diseases and chronic obstructive pulmonary disease and allied cond (COPD) were the most common non-cancer causes of death. By 5 years after PCa diagnosis, the proportion of deaths from cardiovascular diseases exceeded that of deaths from PCa (**Table 3**). Several previous studies using the SEER program have shown that cardiovascular diseases and COPD are the most common non-cancer causes of death in the cancer patients in the United States, especially PCa patients (8, 9, 29). In a cohort study of 15,878 patients from Denmark, the most common non-cancer cause of death in PCa patients was cardiovascular disease, accounting for 17.0% of all deaths (12). There may be several reasons for this significant change. ADT is the main treatment method for patients with metastatic PCa. Studies have shown that patients receiving ADT have a higher incidence of cardiovascular disease and mortality (30, 31), which may be due to its metabolic effects (32). Most patients treated with ADT will progress to hormone-refractory PCa at a later stage. Furthermore, docetaxel is the standard treatment for this group of patients. However, anthracycline chemotherapy drugs have strong cardiotoxicity with a cumulative effect of time and dose (33).

Interestingly, the risk of death from Alzheimer’s disease increased exponentially as the time after diagnosis increased. After more than 10 years since cancer diagnosis, the risk of death from Alzheimer’s disease in PCa patients was more than 20 times that of the cancer-free population (**Table 3**). Previous studies have found that longer ADT treatment is associated with a higher risk of Alzheimer’s disease, but the results are still controversial and require more solid evidence (34, 35). Additionally, Medicare data show that nearly 35% of their 1.2 million PCa patients chose ADT treatment from 2001 to 2014 (30).

At the other extreme, we found that the relative risks of death from suicide and gastroduodenal ulcers in the first year after diagnosis are significantly higher than those 1–5 years after diagnosis (**Table 3**). Psychological trauma is severe for patients in the first year of cancer diagnosis and treatment. Studies have shown that the incidence of clinical depression increases after cancer diagnosis, which may explain this phenomenon (36). As for gastroduodenal ulcers they may be affected by therapeutic drugs. On the other hand, as a psychosomatic disease, psychological shock will increase the incidence and severity of ulcers (37). Therefore, it is necessary to strengthen positive and powerful psychological interventions for newly diagnosed PCa patients to encourage them to correctly understand PCa and accept the facts.

Our study differs from previous studies that used SEER data to analyze causes of death among PCa patients. Zaorsky et al. performed an analysis of causes of death among all cancer patients, which included patients of all ages (8). PCa is known to be a disease of old age, and young patients often have hereditary disease (38). Therefore, excluding patients younger than 40 years in our study could make the results more accurate. In addition, Zaorsky et al. used the calendar year to analyze the temporal trend, but this could not avoid the bias caused by the different lengths of follow-up of patients diagnosed in different years. Our study used age at diagnosis to analyze the temporal trend, which is more accurate. Another study using SEER data did not cover PCa cases in the 20th century (29). Inclusion of cases before the 1990s is necessary to analyze changes in causes of death among PCa patients before and after the adoption of PSA screening (2). In 2011, Riihimäki et al. reported changes in the causes of death in Swedish PCa patients from 1987 to 2006, and they found that the highest risks of non-cancer deaths among Swedish PCa patients were suicide and pulmonary circulation (39). However, our study found that PCa patients in the United States had the highest risk of dying from Alzheimer’s disease and hypertension. This shows that there are large differences in causes of death among PCa populations in different countries.

Our analysis also has some limitations. First, the SEER 18 database (2019 submission) lacks information on patient comorbidities, family history, and complete information on treatment, all of which may have an impact on prognosis; therefore, deeper analysis such as risk stratification and assessment of the impact of ADT is impossible. Second, many cases had unclear values for some variables, such as PSA and Gleason score, which reduces the strength of the evidence in this section. Third, the length of follow-up for patients diagnosed in different years was different. Patients diagnosed earlier have a longer follow-up period for all-cause mortality, and survival with tumors lasts longer. In addition, although the SEER database uses a systematic standard collection procedure to ensure the accuracy of the cause of death, there is still some possibility of reporting bias in death certificates.

## Conclusion

Non-cancer causes have replaced primary cancer as the main cause of death for PCa patients in the United States, and patients with PCa have a higher risk of death than the general population. As the survival time of patients increases, the proportion of non-cancer deaths not only will increase but the relative risk will also increase. In the early stage after diagnosis, the risk of index cancer death and psychosomatic diseases, such as suicide, was relatively high. It is necessary to emphasize the education and psychological counseling of patients. As the survival period extends, non-cancer causes of death such as cardiovascular disease, COPD, and Alzheimer’s disease will dominate. Attention should be paid to lifestyle improvement and the rational use of tumor drugs.

## Data Availability Statement

The original contributions presented in the study are included in the article/supplementary material. Further inquiries can be directed to the corresponding authors.

## Author Contributions

Conception and design: XZ, HR, and YY. Collection and assembly of data: YY and YZ. Data analysis and interpretation: YY, YZ, and QM. Manuscript writing: All authors. Final approval of manuscript:All authors. Accountable for all aspects of the work: All authors.

## Funding

This work was supported by grants from the National Key Scientific Instrument Development Project (81927807), National Key R&D Program of China (2017YFB1303100), National Natural Sciences Foundation of China (82002706).

## Conflict of Interest

The authors declare that the research was conducted in the absence of any commercial or financial relationships that could be construed as a potential conflict of interest.

## Publisher’s Note

All claims expressed in this article are solely those of the authors and do not necessarily represent those of their affiliated organizations, or those of the publisher, the editors and the reviewers. Any product that may be evaluated in this article, or claim that may be made by its manufacturer, is not guaranteed or endorsed by the publisher.

## Abbreviations

SMR, standardized mortality ratio; PCa, prostate cancer; CI, confidence interval; PSA, prostate-specific antigen; SEER, Surveillance, Epidemiology, and End Results; CMR, cumulative mortality rate; ADT, androgen deprivation therapy; COPD, chronic obstructive pulmonary disease and allied cond.
